# Optimization of the Process of Extracting Polysaccharides from *Agrocybe aegerita* and In Vitro Antioxidant and Anti-Aging Tests

**DOI:** 10.3390/molecules29214992

**Published:** 2024-10-22

**Authors:** Yuhan Wang, Jingyi Yang, Xiang Li, Jingshuo Yang, Honglei Wang

**Affiliations:** 1 Yantai Institute of China Agricultural University, Yantai 264670, China; 16678255906@163.com (Y.W.); 15253310311@163.com (J.Y.); 13256729486@163.com (X.L.); 2 College of Food Science and Nutritional Engineering, China Agricultural University, Beijing 100083, China; yang_cubejs0916@163.com

**Keywords:** *Agrocybe aegerita* polysaccharides (AAPs), response surface methodology, antioxidant activity, *Caenorhabditis elegans*, anti-aging activity

## Abstract

The extraction process of crude polysaccharides from *Agrocybe aegerita* was optimized, and the antioxidant and anti-aging effects of the crude polysaccharides were evaluated. The optimal extraction parameters for the polysaccharide were identified using the response surface methodology. The DPPH, hydroxy radical, and superoxide anion radical scavenging capacities were evaluated to determine the antioxidant properties of the AAPs. The effects of the AAPs on the lifespan, head-swing frequency, swallowing frequency, body-bending frequency, and stress resistance of *Caenorhabditis elegans* were determined. The optimal extraction conditions included a solid-to-liquid ratio that resulted in an extraction solution concentration of 0.034 g/mL, an extraction temperature of 92.64 °C, and an extraction time of 2.82 h. Under these conditions, the yield of the AAPs was 11.325% ± 0.996%. The IC50 of the AAPs for superoxide anion radical scavenging was 4.693 mg/mL. The AAPs reached their maximum activity at concentrations of about 2 mg/mL for DPPH and 5 mg/mL for the hydroxyl radical. The AAPs could prolong the lifespan and improve locomotion and the stress resistance of *C. elegans*. Our findings illustrate the potential of AAPs as an anti-aging and antioxidant agent, highlighting the use of this natural compound in the fields of food and pharmacology.

## 1. Introduction

*Agrocybe aegerita* belongs to the *Basidiomycetes* class and the *Strophariaceae* family and is an edible mushroom that is popular in China for being rich in nutrients and its unique flavor. In addition, it has many effects when used as a traditional Chinese medicine, such as invigorating the spleen and diuresis and having antidiarrheal and moistening effects [1]. Polysaccharides from edible fungi are important bioactive substances that can enhance humoral and cellular immunity [2]. Studies have confirmed that many components contained in *A. aegerita*, such as polysaccharides, indole derivatives, etc., have a clear ability to eliminate free radical antioxidants, as well as anti-aging, antitumor, immunity-enhancing, and other biological activities [3,4,5,6].

Aging is a degenerative physiological process characterized by a decline in biological function, adaptability, and resistance, wherein oxidative stress damage is closely related to aging [6]. It is a long-term, irreversible, gradual, and complex physiological process. Delaying the aging process and extending the lifespan is a scientific issue that has attracted immense attention. *Caenorhabditis elegans* has been widely used as a model organism for anti-aging research owing to its desirable characteristics of highly conserved evolution, a short lifecycle, a stable behavioral-response pattern, and sensitive and reliable results. Furthermore, it exhibits aging characteristics similar to those of mammals. Therefore, *C. elegans* is used as a model for studying longevity and determining the physiological functions of polysaccharides from edible fungi [7,8,9].

**Figure 1:**
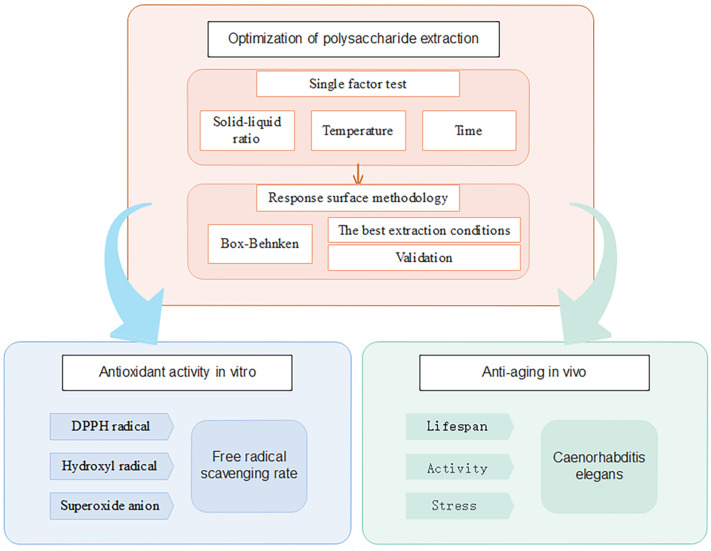
Extraction optimization and the antioxidant and anti-aging activity of the crude polysaccharide from *Agrocybe aegerita*.

In this study, AAPs were obtained using hot-water extraction, and the extraction parameters were optimized using the response surface methodology (RSM), a technology that can be used to scale up AAPs extraction in an industrial setting. The antioxidant activity of the AAPs was determined based on its in vitro scavenging ability for DPPH radicals, hydroxyl radicals, and superoxide anions. The anti-aging effect of the crude AAPs on the lifespan, swallowing frequency, locomotion ability, and stress resistance of *C. elegans* was studied. A flow chart is shown in Figure 1. Our findings provide theoretical support for the further development of AAPs as a functional food owing to their anti-aging and antioxidant effects.

## 2. Results and Discussion

### 2.1. Single-Factor Experiments

#### 2.1.1. Effects of the Solid-to-Liquid Ratio on AAPs Yield

**Figure 2:**
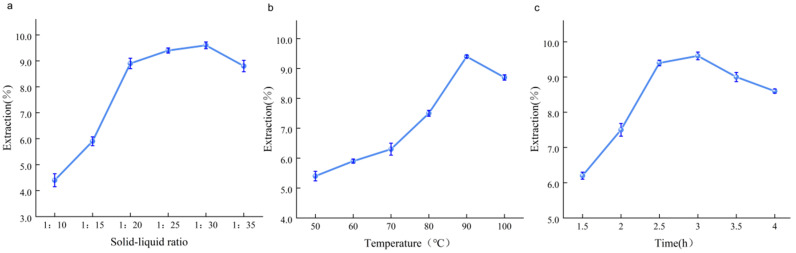
(**a**–**c**) Single-factor test for the extraction of *A. aegerita* polysaccharides.

The solid-to-liquid ratio in the extract may directly affect the concentration of the extract, which, in turn, can lead to varying degrees of inhibition of polysaccharide dissolution [10,11]. Figure 2a shows that an increase in the solid-to-liquid ratio initially increases the AAPs yield significantly and then decreases it, indicating that a higher solid-to-liquid ratio increases the dissolution of AAPs in water. A high amount of polysaccharides and nonsugar substances dissolved when excess solvent was used, thereby affecting the polysaccharide yield [12].

#### 2.1.2. Effects of the Extraction Temperature on AAPs Yield

The polysaccharide yield increased significantly with an increase in extraction temperature in the range of 50–90 °C, indicating that the increase in temperature increased the dissolution rate of the polysaccharide in the *A. aegerita* powder (Figure 2b). When the temperature was further increased, the polysaccharide yield decreased. The reasons for this were as follows: The increase in temperature accelerated molecular movement and rendered the polysaccharide more soluble. This revealed that temperature enhanced the extraction of crude polysaccharides from *A. aegerita* into the water up to a certain level, followed by a possible loss due to decomposition at a higher temperature [13,14].

#### 2.1.3. Effects of Extraction Time on AAPs Yield

The polysaccharide yield increased significantly with an increase in extraction time in the range of 1.5–2.5 h, indicating that the prolongation in extraction time led to increased dissolution of the polysaccharide (Figure 2c). Additionally, no significant increase was noted in the extraction rate of the polysaccharide when the extraction time was increased to 2.5–3 h. The extraction rate of the AAPs decreased as the extraction time increased. A possible reason for this could be that the glucoside bond of the polysaccharide was easily broken during extraction at high temperatures for a prolonged period, resulting in a decreased yield [11,15].

### 2.2. Optimization of Extraction Conditions Using Box–Behnken Design

Table 1 shows the experimental results of the optimization using the RSM. Equation (1) was obtained by regression analysis, and the extraction rate of the AAPs was obtained as follows:

(Y) Y = 11.20 + 0.73A + 0.66B − 0.32C + 0.25AB + 0.095AC − 0.13BC − 0.91A^2^ − 1.90B^2^ − 0.45C^2^ (1)

The results of the analysis of variance are shown in Table 2, *p* < 0.01, which are extremely significant, indicating that the model is effective and reliable. The *p*-value for the lack of fit term was 0.7896, which is greater than 0.05 and therefore not significant, indicating that there was no obvious lack of fit factor, which could be used as a key point in the experiment for analysis. The coefficient of determination (R^2^) was 0.9945, and the coefficient of variance was 1.59%, indicating that the equation had good fit and stability as well as high accuracy. The F-value revealed that the effects of the three factors on the extraction rate of the polysaccharides were in the order of B > A > C, and all values were extremely significant (*p* < 0.01).

**Figure 3:**
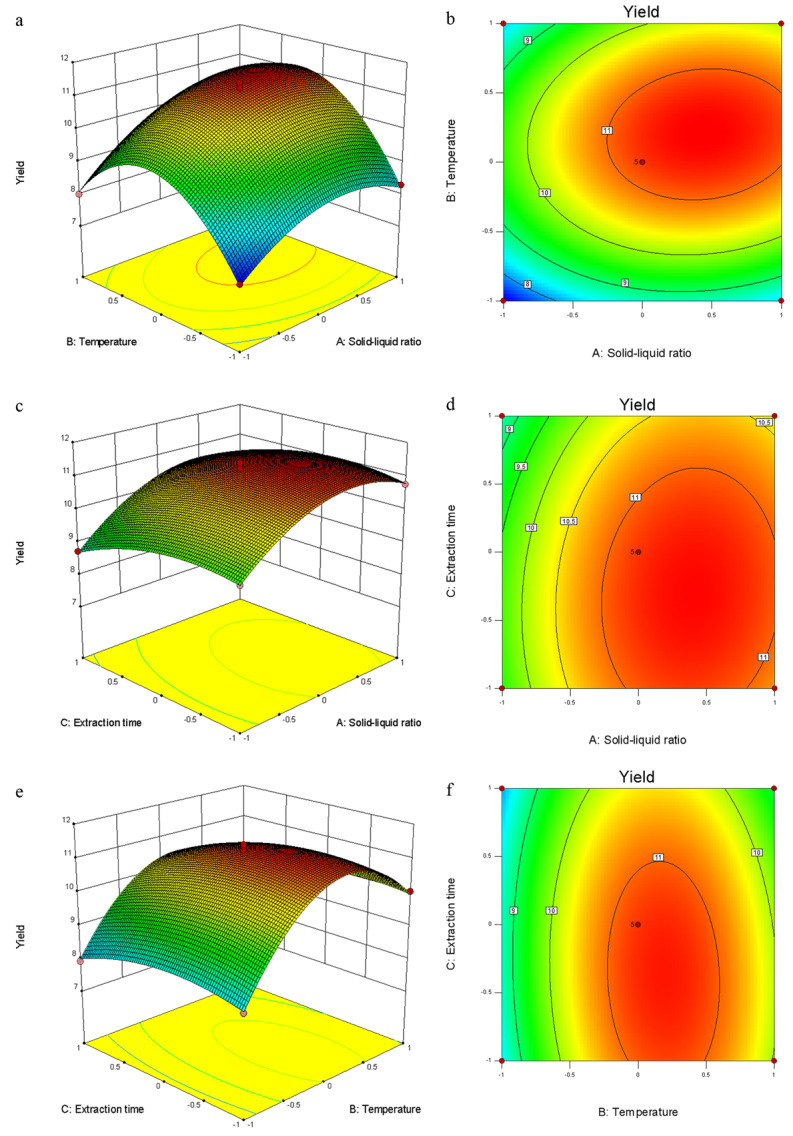
(**a**–**f**) Response surface methodology used to determine the interaction effects of various factors on the extraction yield of AAPs. (A–C) Response surface plots of solid-to-liquid ratio (X_1_, g/mL), extraction temperature (X_2_, °C), and extraction time (X_3_, h) on the extraction yield of AAPs.

The three-dimensional response surface curve reflects the influence of the interaction between factors on the AAPs extraction rate. The larger the slope, the greater the impact, and vice versa. The results are shown in Figure 3, where the slope of the solid-to-liquid ratio and extraction temperature is the greatest, indicating the interaction between them to be the largest, followed by extraction temperature and extraction time. These findings are consistent with the results presented in Table 1.

### 2.3. Verification of the Optimal Extraction Process

The predicted optimal process conditions were as follows: the solid-to-liquid ratio concentration was 0.034 g/mL, the extraction temperature was 92.64 °C, the extraction time was 2.82 h, and the extraction rate of polysaccharides was predicted to be 11.467%. After correction based on the predicted optimal process conditions, the solid-to-liquid ratio was 1:29, the extraction temperature was 93 °C, and the extraction time was 2.8 h. Using these conditions, three experiments were performed for the extraction of AAPs. The actual extraction rate of the polysaccharides was 11.325% ± 0.996%, indicate that the prediction model was reliable.

### 2.4. Antioxidant Capacity

#### 2.4.1. DPPH Radical Scavenging Capacity of AAPs

**Figure 4:**
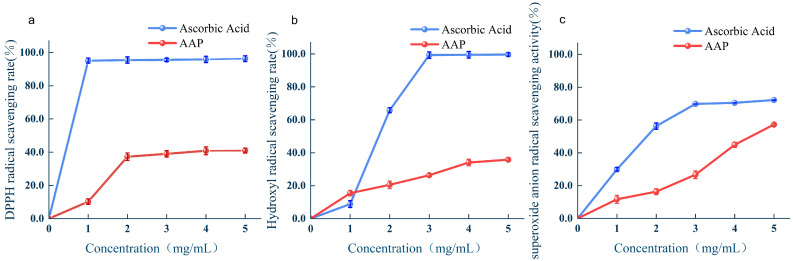
(**a**–**c**) Effects of AAPs on scavenging DPPH, hydroxyl, and superoxide anion radicals.

The effect of different AAPs concentrations on DPPH free radical scavenging is shown in Figure 4a. At low concentrations, the DPPH radical scavenging rate was positively correlated with polysaccharide concentration. At high AAPs concentrations, the DPPH scavenging rate was stable and showed an increasing but slow trend, which may be because the polysaccharide molecules repelled each other, leading to a decrease in the ability to capture free radicals as the concentration increased [16,17]. When the concentration of polysaccharides was 5 mg/mL, the DPPH free radical scavenging rate reached 40.85% ± 0.54%.

#### 2.4.2. Hydroxyl Radical Scavenging Capacity of AAPs

The effect of different concentrations of AAPs on hydroxyl radical scavenging is shown in Figure 4b. The hydroxyl radical scavenging ability of the AAPs was positively correlated with their concentration. At a concentration of 5 mg/mL, the hydroxyl radical scavenging rate was 35.78% ± 0.60%. At a concentration of 1 mg/mL, the hydroxyl radical scavenging activity of the polysaccharides was stronger than that of ascorbic acid.

#### 2.4.3. Superoxide Anion Radical Scavenging Capacity of AAPs

The superoxide anion radical scavenging ability of the AAPs was positively correlated with AAPs concentration (Figure 4c). At a concentration of 4 mg/mL, the superoxide anion radical scavenging rate of the AAPs was 44.90% ± 1.57%.

### 2.5. Anti-Aging Capacity

#### 2.5.1. Effect of AAPs on the Lifespan of *C. elegans*

**Figure 5:**
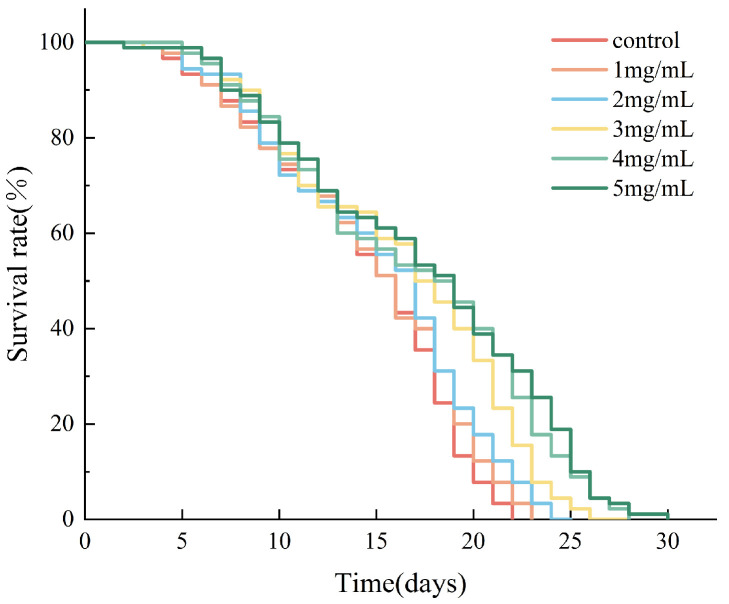
Survival curves of *C. elegans* treated with AAPs (0, 1, 2, 3, 4, and 5 mg/mL).

The lifespan of *C. elegans* can be used as a quantitative index of senescence [18,19]. The effects of AAPs on the lifespan of *C. elegans* are shown in Figure 5 and Table 3. The mean, median, and longest lifespan of *C. elegans* in the polysaccharide-treated group were higher than those of *C. elegans* in the control group. The survival curve shifted to the right with an increase in AAPs concentration. These results indicated that AAPs could delay the natural aging of *C. elegans* and that this effect could be enhanced with an increase in AAPs concentration within a certain concentration range.

#### 2.5.2. Effect of AAPs on the Movement and Swallowing Abilities of *C. Elegans*

Changes in movement and swallowing abilities affect the vitality of *C. elegans*. Movement behavior can indicate the physical function and senescence of the body, whereas bending frequency and head-swing frequency are basic indices indicative of the locomotion ability of *C. elegans*. The swallowing frequency of *C. elegans* decreases with age and is related to its food-intake ability.

**Figure 6:**
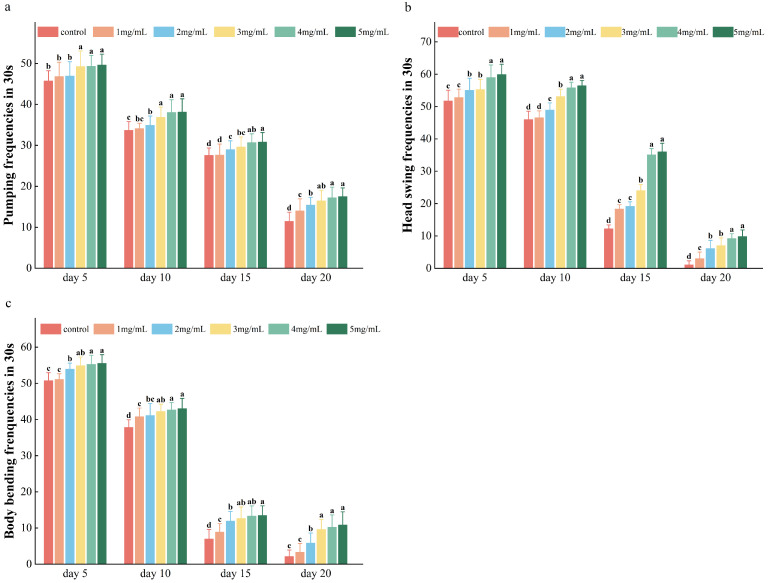
(**a**–**c**) Effects of AAPs on the swallowing frequency, head-swing frequency, and body-bending frequency of *C. elegans*. The data are expressed as the means ± standard deviations; the different letters on the bars (a, b and c) indicate statistically significant differences in all of the treatment groups (*p* < 0.05).

The swallowing frequency of *C. elegans* was significantly increased after polysaccharide treatment on days 15 and 20, indicating the effect of the polysaccharides in the middle and later stages. The AAPs also exerted a certain inhibitory effect on the senescence of *C. elegans* (Figure 6a, *p* < 0.05). Furthermore, the AAPs were able to significantly increase the head-swing frequency (Figure 6b, *p* < 0.05) and body-bending frequency (Figure 6c, *p* < 0.05) of *C. elegans*. These findings suggest that AAPs intervention can increase the swallowing, head-oscillation, and body-bending frequencies of *C. elegans*.

#### 2.5.3. Effect of AAPs on Stress Resistance

**Figure 7:**
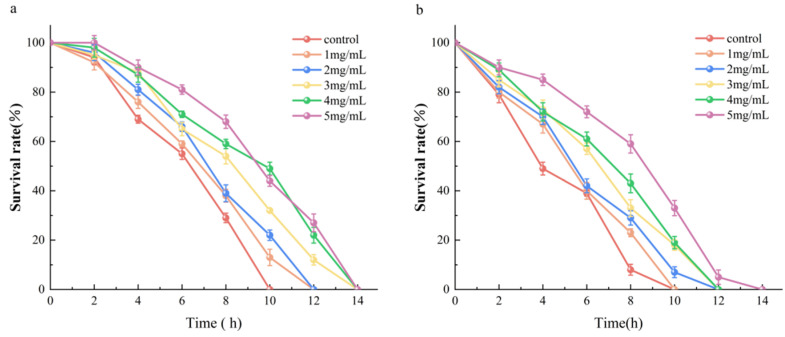
Survival curves of *C. elegans* treated with AAPs (0, 1, 2, 3, 4, and 5 mg/mL) after heat stress (37 °C) (**a**) and juglone stimulus (**b**).

The prolongation of the lifespan has a strong correlation with the enhancement of stress. High temperatures can cause collective metabolic disorder, thereby producing a large number of reactive oxygen species and resulting in oxidative stress [20,21]. Therefore, the resistance of nematodes to acute oxidative stress was investigated by inducing heat stress. The lifespan of *C. elegans* under heat stress at 37 °C is shown in Figure 7a. The average survival time of *C. elegans* in the control group was 4.87 h. Different concentrations of AAPs had varying effects on the survival time of *C. elegans*, and the average lifespan of *C. elegans* in the experimental group was extended by 21.15%. The AAPs at concentrations of 4 mg/mL and 5 mg/mL significantly prolonged the survival time of *C. elegans* (*p* < 0.05), and the survival curve shifted to the right. These results indicated that AAPs could protect nematodes from heat stress injury, improve their ability to resist heat stress, prolong their lifespan, and improve their physiological health index.

Juglone was chosen as a strong oxidant to establish an environment of oxidative damage. As shown in Figure 7b, the average survival time of *C. elegans* in the control group was 3.65 h. The survival time was prolonged when different concentrations of AAPs were used. The average lifespan of *C. elegans* in the experimental group was prolonged by 29.04%. The AAPs at concentrations of 3 mg/mL, 4 mg/mL, and 5 mg/mL significantly prolonged the survival time (*p* < 0.05), and the survival curve shifted to the right. These findings depicted that AAPs could be used as plant-derived antioxidants to enhance the resistance of *C. elegans*, repair oxidative damage, and exert a beneficial antioxidant effect. Overall, AAPs could increase the tolerance of *C. elegans* to juglone.

## 3. Materials and Methods

### 3.1. Chemicals and Reagents

*C. elegans* and *Escherichia coli* OP50 were provided by the School of Food Science and Nutrition Engineering, China Agricultural University, Beijing, China. *A. aegerita* was purchased from Qingyuan Alpine agricultural products, Lishui, China. LB broth, LB agar, and Nematode Growth Medium (NGM) were purchased from Shandong Topu Bioengineering Co., Ltd., Yantai, China. Anhydrous ethanol was purchased from Sinopharm Chemical Reagent Co., Ltd., Shanghai, China. The DPPH radical scavenging kit, hydroxyl radical scavenging kit, and superoxide anion radical scavenging kit were procured from Suzhou Geruis Biotechnology Co., Ltd., Suzhou, China.

### 3.2. Instruments and Equipment

An H2050r centrifuge was obtained from Hunan Xiangyi Laboratory Instrument Development Co., Ltd., Hunan, China. A Yp1002n electronic balance was obtained from Shanghai Precision Scientific Instrument Co., Ltd., Shanghai, China. An Evolution300 UV spectrophotometer was purchased from Thermo Fisher Scientific, Inc, Shanghai, China. A Sci-vs vortex mixer was obtained from Coetzee scientific laboratory, Yantai, China. A BHS-4 digital display thermostatic water bath was purchased from Jiangyin Poly Scientific Research Instruments Co., Ltd., Jiangyin, China. A Dhg-9053a electric-heating constant-temperature blast drying oven was obtained from Shanghai Yiheng Scientific Instrument Co., Ltd., Shanghai, China. A Bsd-yx2400 vertical double-layer rocker was purchased from Shanghai Boxun Industrial Co., Ltd., Shanghai, China.

### 3.3. Extraction of AAPs

*A. aegerita* was crushed and sieved through a 100-mesh sieve to obtain a powder. The powder was weighed, and water was added according to the set solid-to-liquid ratio. After stirring to form a suspension, the powder was allowed to soak for 20 min and then extracted using the solid-to-liquid ratio, extraction temperature, and extraction time chosen in this study. After centrifugation at 5000 rpm for 15 min, anhydrous ethanol was added to the supernatant until the ethanol concentration was 85%. The resulting solution was placed in a refrigerator at 4 °C for alcoholysis for 24 h. After centrifugation at 5000 rpm for 15 min, the obtained solid was dried in an oven at 60 °C for 24 h and ground to a powder to obtain crude AAPs. The yield of the AAPs was calculated using the following formula:

Z (%,*w*/*w*) = Y/Y_0_ × 100 (2)

where Z is the yield of AAPs, Y is the mass of AAPs obtained after drying, and Y_0_ is the mass of *A. aegerita* powder.

### 3.4. Optimization of Polysaccharide Extraction

#### 3.4.1. Single-Factor Experiments

Taking the yield of the AAPs as the index, the effects of different ratios of the extraction solvent as well as the effects of extraction temperature and extraction time on the extraction efficiency of AAPs were studied [22,23,24,25]. (1) The extraction time was set at 2.5 h, the extraction temperature was set at 90 °C, and the effects of solid-to-liquid ratios (g:mL) of 1:10, 1:15, 1:20, 1:25, 1:30, and 1:35 on the extraction rate of AAPs were studied. (2) The extraction time was set at 2.5 h, the solid-to-liquid ratio was set at 1:25, and the effects of extraction temperatures of 50 °C, 60 °C, 70 °C, 80 °C, 90 °C, and 100 °C on the extraction rate of AAPs were studied. (3) The extraction temperature was set at 90 °C, the solid-to-liquid ratio was set at 1:25, and the effects of extraction times of 1.5 h, 2 h, 2.5 h, 3 h, 3.5 h, and 4 h were evaluated.

#### 3.4.2. Response Surface Methodology

Based on single-factor experiments, the extraction parameters for AAPs were optimized using an RSM Box–Behnken design with 3 factors, namely the solid-to-liquid ratio, extraction temperature, and extraction time (Table 4). A regression model was established to predict the optimal combination of these extraction process parameters. Lastly, the prediction results of the model were verified, and the optimal extraction parameters were obtained [26,27,28].

### 3.5. Antioxidant Activities In Vitro

#### 3.5.1. Determination of DPPH Free Radical Scavenging Rate

The DPPH free radical scavenging rate was calculated according to the instructions in the kit. In this assay, 100 μL of AAPs at concentrations ranging from 1 to 5 mg/mL [24] was mixed with 600 μL of DPPH solution (1.0 mmol/L). The mixture was thoroughly agitated and left to stand for 30 min in the dark at 25 °C, followed by an absorbance measurement at 517 nm, with distilled water used as a negative control and ascorbic acid as a positive control. The DPPH radical scavenging activity was calculated using the following equation:

(3) $$\mathrm{Scavenging}\;\mathrm{rate}\;(\%)=(1-\frac{A_{1}-A_{2}}{A_{0}})\times 100$$

A_1_: the absorbance value recorded for the sample solution.

A_2_: the absorbance value recorded for the background solution, which was prepared using 80% methanol instead of the DPPH solution.

A_0_: the absorbance value recorded for the background solution, which was prepared using 80% methanol instead of the sample solution.

#### 3.5.2. Determination of Hydroxyl Radical Scavenging Rate

The hydroxyl radical scavenging rate was calculated according to the instructions in the kit. In this assay, 125 μL of AAPs at concentrations ranging from 1 to 5 mg/mL [24] was mixed with an equal volume of FeSO4 solution (9.0 mmol/L) and a salicylic acid solution in absolute ethanol (9.0 mmol/L). This mixture was then reacted with 125 µL of H_2_O_2_ solution (8.8 mmol/L) in a reaction tube maintained at 37 °C for 20 min. If there was turbidity in the measuring tube, it was centrifuged for 5 min at room temperature and 8000 rpm, all the clarified liquid was transferred to a 1 mL glass colorimetric dish, and the absorption value of each tube was immediately read at 510 nm, with distilled water as a negative control and ascorbic acid as a positive control. The hydroxyl radical scavenging activity was calculated using the following equation:

(4) $$\mathrm{Scavenging}\;\mathrm{rate}\;(\%)=(1-\frac{A_{1}-A_{2}}{A_{0}})\times 100$$

A_1_: the absorbance value recorded for the sample solution.

A_2_: the absorbance value recorded for the background solution, which was prepared using distilled water instead of H_2_O_2_ solution.

A_0_: the absorbance value recorded for the background solution, which was prepared using distilled water instead of the sample solution.

#### 3.5.3. Determination of Superoxide Anion Radical Scavenging Rate

The superoxide anion radical scavenging rate was calculated according to the instructions in the kit. In this assay, 40 μL of AAPs at concentrations ranging from 1 to 5 mg/mL [24] was mixed with 500 μL of 100 mmol/L phosphate buffer, 160 μL of a concentration of 300 μmol/L NBT, and 40 μL of NADH at a concentration of 940 μmol/L. This mixture was then reacted with 60 μL of PMS at a concentration of 120 μmol/L in a reaction tube maintained at 37 °C for 10 min, followed by absorbance measurement at 570 nm, with 100 mmol/L phosphate buffer as a negative control and ascorbic acid as a positive control. The scavenging activity of superoxide anion radicals was calculated using the following equation:

(5) $$\mathrm{Scavenging}\;\mathrm{rate}\;(\%)=(1-\frac{A_{1}-A_{2}}{A_{0}})\times 100$$

A_1_: the absorbance value recorded for the sample solution.

A_2_: the absorbance value recorded for the background solution, which was prepared using 100 mmol/L phosphate buffer instead of NADH solution.

A_0_: the absorbance value recorded for the background solution, which was prepared using 100 mmol/L phosphate buffer instead of the sample solution.

### 3.6. Anti-Aging Effect

#### 3.6.1. Synchronization Processing

*C. elegans* was grown on NGM coated with *Escherichia coli* OP50. When there were many adults in the spawning period, *C. elegans* were washed with 1 mL of M9 buffer in a sterile Eppendorf tube and centrifuged for 30 s at 10,000 rpm. The supernatant was discarded, and 1 mL of lysate was added, vortex-oscillated for 3 min, and centrifuged again for 30 s. The supernatant was discarded, and 1 mL of M9 buffer was added to the precipitate and washed for 5 min with vortex oscillation, washed 3 times, and centrifuged, following which the supernatant was discarded. The nematode suspension drops were absorbed in NGM coated with OP50 and cultured at 20 °C for approximately 48 h (no more than 60 h) to obtain L4 larvae [29,30,31].

#### 3.6.2. Experimental Treatment

Synchronized L4-stage *C. elegans* were randomly selected and cultured at 20 °C on an OP50-NGM plate coated with 200 μL of AAPs solution with mass concentrations of 0, 1, 2, 3, 4, and 5 mg/mL. The lifespan, swallowing frequency, exercise ability, and stress resistance of *C. elegans* were measured.

#### 3.6.3. Lifespan Assay

*C. elegans* were transferred to a new corresponding medium plate daily, and their survival and death were recorded until all *C. elegans* died. No response to 2 stimuli was used as the standard to determine death (30 *C. elegans* per plate, 3 per group) [32,33,34].

#### 3.6.4. Evaluation of *C. elegans* Activity

The head-swing frequency, body-bending frequency, and swallowing ability of nematodes were measured on the 5th, 10th, 15th, and 20th days, whereas the exercise ability was measured on the 6th, 12th, and 18th days. (1) Measurement of swallowing ability: the suction frequency of the terminal pharyngeal bulb of each nematode was observed and recorded within 30 s by counting using a microscope (30 pieces per group) [35]. (2) Determination of head-swing frequency: one nematode was randomly chosen and put on a blank NGM plate containing 1 mL of M9 buffer. After adaptation for 30 s, the head-swing frequency was measured within 30 s. Head-swing behavior was defined as the change in the head-swing direction with a change angle of more than 90° (30 *C. elegans* per group) [36]. (3) Measurement of body-bending frequency: the number of instances of body bending in nematodes within 30 s was recorded. Body bending was defined by considering the direction along the pharyngeal pump as the Y-axis. While the *C. elegans* were crawling around, a change in the direction along the corresponding X-axis was considered body bending (30 *C. elegans* per group) [36].

#### 3.6.5. Stress Assay

After 6 days of culture, 30 *C. elegans* were randomly selected from each group for heat stress and oxidative stress experiments. Five parallel experiments were conducted for each experiment, and the average lifespan of each group was calculated.

#### 3.6.6. Thermal Stress

*C. elegans* were transferred to blank NGM and cultured at 37 °C at a constant temperature. The survival number of *C. elegans* was observed and recorded every 2 h until all *C. elegans* in the dish were dead [37,38].

#### 3.6.7. Oxidative Stress

*C. elegans* were transferred to NGM containing a final juglone concentration of 400 μmol/L without OP50, and the number of *C. elegans* was observed and recorded every hour until all *C. elegans* on the dish were alive [39,40].

### 3.7. Design Experiment Software

Each experiment was performed more than 3 times. Data are expressed as mean ± standard deviation. IBM SPSS Statistics 26 was used to analyze the significant differences in the experimental data. *p* < 0.05 indicates a significant difference at the 0.05 level. The RSM was designed by Design Expert. Experimental data from each group were analyzed using OriginPro 2024b.

## 4. Conclusions

In this survey, the RSM was used to optimize operational parameters (a solid-to-liquid ratio that resulted in an extraction solution concentration of 0.034 g/mL, a temperature of 92.64 °C, and an extraction time of 2.82 h) to obtain the maximum yield (11.325% ± 0.996%) of AAPs. The RSM was designed using the Box–Behnken design to systematically analyze the effects of extraction parameters and their interactions on the yield of AAPs. Our findings could guide the process of scaling up AAPs extraction in industrial settings.

The antioxidant activity of the crude AAPs was evaluated based on DPPH radical, hydroxyl radical, and superoxide anion radical scavenging experiments. Our findings revealed that the scavenging of these free radicals was positively correlated with the AAPs concentration. The IC50 of the AAPs for superoxide anion radical scavenging was 4.693 mg/mL. The AAPs reached their maximum activity at concentrations of about 2 mg/mL for DPPH and 5 mg/mL the hydroxyl radical. The results from the in vitro experiments showed the dose-dependent antioxidant effect of AAPs. The model organism *C. elegans* was used to evaluate the anti-aging effect of the AAPs. Anti-aging indices of *C. elegans* such as swallowing frequency, locomotion ability, and resistance to juglone and heat stress increased significantly, and lifespan was significantly prolonged, indicating the significant anti-aging effect of AAPs. Liu’s findings revealed that AAPs extended the lifespan of naturally aging D. melanogaster and mice and improved their resistance to stress. These experimental results illustrated the remarkable antioxidant and anti-aging properties of AAPs. This is consistent with our findings [6]. These findings suggest AAPs as a promising natural anti-aging agent and highlight their potential for use in scientific and theoretical development and utilization in food, medicine, and cosmetics. However, the specific molecular mechanism of the anti-aging effect of AAPs needs to be further evaluated. Furthermore, findings in the model organism *C. elegans* are still limited. Further confirmation of the anti-aging effect of AAPs in mammals and based on clinical trials is therefore warranted.

## Figures and Tables

**Table 1:** Design and results from response surface methodology on the extraction of AAPs.

Test Number	A/Extraction Temperature (°C)	B/Extraction Time (min)	C/Extraction Ratio (mg/mL)	Extraction Rate (%)
1	−1	−1	0	7.302
2	1	−1	0	8.297
3	−1	1	0	8.004
4	1	1	0	9.982
5	−1	0	−1	9.524
6	1	0	−1	10.762
7	−1	0	1	8.731
8	1	0	1	10.351
9	0	−1	−1	8.341
10	0	1	−1	10.036
11	0	−1	1	7.924
12	0	1	1	9.112
13	0	0	0	11.321
14	0	0	0	10.952
15	0	0	0	11.402
16	0	0	0	11.087
17	0	0	0	11.231

**Table 2:** Result of analysis of variance using the Box–Behnken design.

Source of Variance	Sum of Squares	Degrees of Freedom	Mean Square	F-Value	*p*-Value
Model	29.83	9	3.31	140.27	<0.0001
A	4.25	1	4.25	179.85	<0.0001
B	3.47	1	3.47	146.91	<0.0001
C	0.81	1	0.81	34.26	0.0006
OFF	0.24	1	0.24	10.22	0.0151
AC	0.036	1	0.036	1.54	0.2541
BC	0.064	1	0.064	2.72	0.1431
A	3.46	1	3.46	146.51	<0.0001
B	15.13	1	15.13	640.20	<0.0001
C	0.85	1	0.85	36.05	0.0005
Residual	0.17	7	0.024	—	—
Lack of Fit	0.035	3	0.012	0.35	0.7896
Pure Error	0.13	4	0.033	—	—
Cor Total	30.00	16	—	—	—

**Table 3:** Statistical table indicating the survival time of *C. elegans* treated with AAPs (0, 1, 2, 3, 4, and 5 mg/mL). The data are expressed as the means ± standard deviations; the different letters (a, b and c) indicate statistically significant differences in all of the treatment groups (*p* < 0.05).

Group	Average Lifespan (Days)	Median Lifespan (Days)	Maximum Lifespan (Days)
0 mg/mL	14.31 ± 1.46 c	16.50 ± 1.00 c	21.67 ± 0.58 d
1 mg/mL	14.66 ± 0.41 c	16.67 ± 0.58 c	23.67 ± 1.15 c
2 mg/mL	15.22 ± 0.38 bc	17.83 ± 0.29 bc	24.67 ± 0.58 c
3 mg/mL	16.71 ± 0.63 ab	19.17 ± 0.76 ab	27.33 ± 0.58 b
4 mg/mL	17.53 ± 0.87 a	19.67 ± 1.26 a	29.67 ± 1.00 a
5 mg/mL	17.80 ± 1.30 a	20.17 ± 1.15 a	30.00 ± 1.73 a

**Table 4:** Response surface analysis factors and levels.

Level		Factor	
	A: Extraction Temperature (°C)	B: Extraction Time (min)	C: Solid-to-Liquid Ratio
−1 0	80 90	150 180	1:35 1:30
1	100	210	1:25

## Data Availability

The raw data supporting the conclusions of this article will be made available by the authors on request.
